# Rab25 Small GTPase Mediates Secretion of Tumor Necrosis Factor Receptor Superfamily Member 11b (osteoprotegerin) Protecting Cancer Cells from Effects of TRAIL

**DOI:** 10.4172/2157-7412.1000153

**Published:** 2013-06-20

**Authors:** KW Cheng, R Agarwal, S Mitra, GB Mills

**Affiliations:** 1 Department of Systems Biology, the University of Texas MD Anderson Cancer Center, Houston, Texas, USA; 2 Department of Surgery & Cancer, Institute of Reproductive and Developmental Biology, Imperial College London, London, W12 0NN, UK

**Keywords:** Osteoprotegerin, Cancer development, Progression

## Abstract

**Background:**

Expression of Rab25, which is located in the 1q amplicon present at high frequency in many cancer lineages, promotes cancer cell survival under multiple stress conditions. While Rab proteins play essential roles in all stages of vesicle trafficking, the functions and endogenous cargoes for Rab25 remain to be fully elucidated. Osteoprotegerin (OPG) is a secreted glycoprotein that binds the tumor necrosis factor-related apoptosis-inducing ligand (TRAIL) thus preventing it from activating the TNF-family death receptors. In the present study, we demonstrated that Rab25 regulates OPG at both the transcription and secretion level.

**Methods:**

The effect of Rab25 on OPG expression and its effect on TRAIL-induced cell were examined in both ovarian and breast cells. Signal transduction pathways regulation of OPG expression was examined in cells using pharmacogenetic approaches.

**Results:**

Expression of Rab25 to levels similar to those in tumors with *RAB25* amplification, increased OPG mRNA expression and secretion from ovarian and breast cancer cell lines, whereas down regulation with Rab25 specific siRNA decreased OPG secretion and sensitized cells to TRAIL-induced cell death. Critically, exogenous OPG mimicked the effects of Rab25 on cell death supporting the contention that Rab25-induced accumulation of OPG protects cancer cells from the effects of TRAIL. Rab25 cooperates with EGFR-mediated MAPK signaling to increase TRAIL production and release. Importantly, priming cells with EGFR inhibitors increased sensitivity to TRAIL-induced cells death regardless of the Rab25 background.

**Conclusion:**

Increased OPG expression induced by Rab25 may provide a mechanistic advantage for cancer development and progression.

## Introduction

Solid tumors are frequently infiltrated by immune cells, including T- and B-lymphocytes, natural killer (NK) cells, NK-T cells, dendritic cells, macrophages, neutrophils, eosinophils and mast cells, that contribute to the tumor microenvironment [1]. Indeed, infiltration with CD8^+^ T cells and a higher ratio of CD8^+^ to CD4^+^ T cell in tumor are associated with an improved outcome and decreased metastasis in multiple cancer lineages indicating that infiltrating immune cells can have anti-tumor activity [2–10].

Cytotoxic CD8^+^ T cells and natural killer cells induce cell death, at least in part, through activation of the extrinsic apoptosis pathway upon binding of FasL (also known as CD95L) and TNF-related apoptosis-inducing ligand (TRAIL), members of the tumor necrosis factor (TNF) family, to death receptors (DR) at the cell surface [1, 11]. FasL and TRAIL trigger apoptosis through binding to Fas (CD95) and DR4 (TRAIL-R1) and/or DR5 (TRAIL-R2) respectively. Upon binding, the death receptors recruit the adaptor molecule Fas-associated death domain (FADD), and the apoptosis-initiating protease caspase-8 into a death-inducing signaling complex (DISC), which in turn engages the intrinsic apoptosis pathway [12]. Both FasL and TRAIL have entered clinical trials with limited efficacy. Cancer cells can acquire resistance to TRAIL-induced apoptosis through multiple mechanisms including loss of functional DR4 and DR5 at the cell surface [13], O-glycosylation status [14], and elevated expression of antiapoptotic proteins including c-FLIP [15, 16], Bcl-2 [16], or IAP family proteins [17].

Tumor necrosis factor receptor superfamily member 11b, also known as osteoprotegerin (OPG), is a secreted glycoprotein belonging to the TNF receptor superfamily that plays a key role in the regulation of bone turnover via acting as a decoy receptor for RANKL and preventing interaction of RANKL with the RANK receptor [18]. In addition to its role in bone metabolism, OPG can also maintain cell survival by acting as a soluble decoy receptor for TRAIL and preventing its interaction with DR4 and DR5 death receptor [19, 20]. The importance of OPG–TRAIL interactions in resistance to TRAIL is underscored by the finding that at physiological conditions OPG can bind TRAIL with an affinity similar to that of RANKL [21]. Hence, release of OPG by tumor cells represents a potential mechanism of resistance to TRAIL-induced apoptosis.

Bypass of cell death mechanisms represents one of the hallmarks of cancer [22]. Thus, an understanding of the mechanisms underlying bypass of cell death could lead to new therapeutic approaches aimed at killing cancer cells. Expression of the Rab25 small G protein, which is a target of the 1q amplicon in multiple cancer lineages, is sufficient to increase cell survival under stress conditions including nutrient withdrawal, anoikis, UV-radiation, and paclitaxel [23, 24]. In the present study, we demonstrate that Rab25 protects tumor cells from death induced by TRAIL, but not FasL, through the production and action of OPG.

## Materials and Methods

### Cells and culture conditions

The human ovarian HEY, SKOV3 and breast MCF7 cells were maintained in RPMI 1640 medium supplemented with 5% fetal bovine serum 5% CO2 at 37°C. IOSE80ht cells were maintained in 1:1 ratio of M199: MCDB105. Rab25 expressing cells and pcDNA empty vector transfected cells were established as described previously [23, 24]. Cells were treated with the indicated concentration of FasL or TRAIL for 24 h. Cells were pretreated with PI3K/AKT or MAPK inhibitors 30min before addition of TRAIL. For EGFR inhibitor studies cells were cultured in the present of Lapatinib, Gefitinib or Neratinib for 60min before addition of TRAIL, FasL or EGF.

### Reagents

FasL (Cat #S8689) was obtained from Sigma and reconstitute in 50 μl of water to a 100 μg/ml stock. Further dilution (working concentration 200 ng/ml) was made with cell culture median containing 5% FBS. Recombinant OPG (Cat #805-OS) and Human Osteoprotegerin/TNFRSF11B ELIZA kit were purchased from R&D systems. Human recombinant TRAIL was purchased from Calbiochem (San Diego, CA), dissolved in phosphate-buffered saline at 100 μg/ml, and stored at ∓80°C. ZD1839 (Gefitinib, Iressa; cat # S1025), Lapatinib (Cat # S2111) and Neratinib (HKI-272; cat# S2150), PI103 (Cat # S1038), PD98059 (Cat # S1177), AG1478 (Cat # S2728) and U0126 (Cat # 1102) were purchased from Selleckchem (Houston, TX). EGF (E9644), TGFα (T7924), IGF (I3769) and PDGF (P8147) were purchased from Sigma Chemical Co. (St. Louis, MO). All reagents were diluted in fresh media before each experiment. On Target Plus siRNA specific to Rab25 and OPG as well as non-target siRNA control were purchased from Dharmacon (Thermo Scientific). siRNA transfection was carried out using DharmaFECT reagent (Thermo Scientific) following manufacturer suggested protocol. Gene expression level after siRNA knockdown was measured by either western blotting or qPCR as reported previously [23–25].

### Cell death assays

Apoptosis cells were determined by Cell Death Dectetion ELISA^plus^ (Cat# 1774425; Roche Apploied Science) according to the manufacturer protocol by measuring the optical density (OD) reading. Cell viability was detected using Cell Titre-Blue^®^ Cell Viability Assay obtained from Promega (Madison, WI).

### Messenger RNA and protein expression

Total RNA isolated was carried out using Qiagen RNeasy kit (Valencia, CA). We determined OPG and Rab25 mRNA levels by Taqman real-time reverse transcription-PCR using the ABI PRISM 7700 Sequence Detection System (Applied Biosystems) through 40 cycles. GAPDH was used as internal reference for Rab25 expression calculation and total RNA quality. Western blotting analysis was carried out as described previously [23, 25]. Antibody against 2238 Phospho-EGF Receptor (Ser1046/1047) (Cat #$2238S), EGF receptor (Cat#2085S), Phospho-Akt (Ser473) (Cat#4058S), Akt (pan) (11E7) Rabbit mAb (cvat#4685S), Phospho-p44/42 MAPK (Erk1/2) (Thr202/Tyr204) (D13.14.4E) XP^®^ Rabbit mAb (Cat#4370S), and total p44/42 MAPK (Erk1/2) (137F5) Rabbit mAb (Cat#4695S) were purchased from Cell Signaling Technology (Danvers, MA).

### Statistical analysis

Experimental data obtained were statistically evaluated by ANOVA or Student’s *t*-test using GraphPad Prism V.5 (San Diego, CA). Differences were considered significant if p<0.05. All experiments were independently repeated at least three times. Data are expressed as mean ± SD of represented experiment.

## Results

### OPG expression decrease TRAIL induced cell death

**Figure 1:**
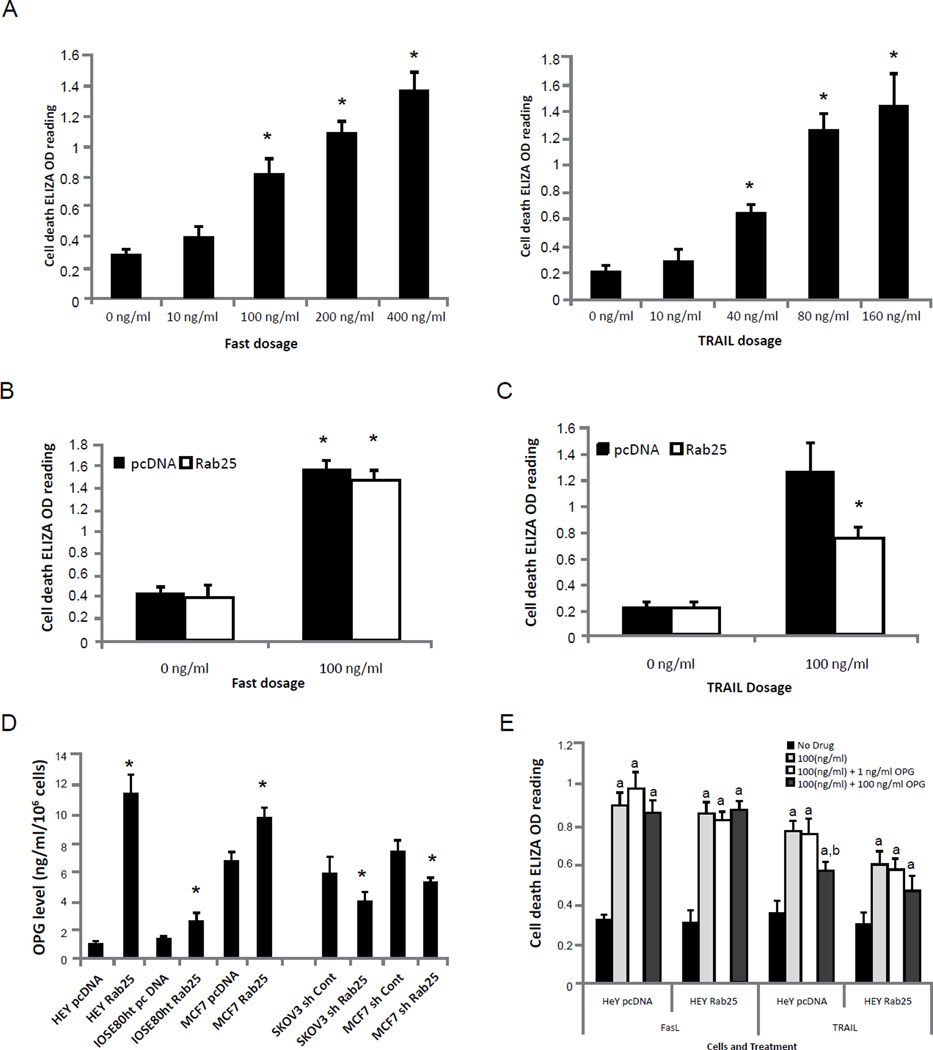
Rab25 expression alters cell sensitivity to TRAIL induced cell death. A) dose-dependent induction of cell death by FasL (left panel) and TRAIL (right panel) in ovarian HEY parental cells, *, p < 0.05 vs 0 ng/ml control. Ovarian HEY cells (1× 10^4^) were treated with FasL or TRAIL with indicated dosage for 24h before detection of apoptosis. Effect of Rab25 expression on cell sensitivity toward (B) 100 ng/ml of FasL (*, p < 0.05 vs 0 ng/ml control) or (C) 100 ng/ml of TRAIL induced cell death (*, p < 0.05 vs pcDNA control). D) Rab25 expression promoted OPG secretion. *, p < 0.05 vs pcDNA control. E) Addition of exogenous OPG blocks TRAIL-induced cell death. a, p < 0.05 vs no drug treatment. b, p < 0.05 vs 100 ng/ml TRAIL treatment.

A dose-dependent study revealed that parental ovarian cancer HEY cell (express very low level of endogenous Rab25^23^ was sensitive to both FasL and TRAIL (Figure 1A). Expression of Rab25, to the level present in patient tumors with amplified *RAB25*, did not alter sensitivity of HEY cells to FasL-induced cell death (Figure 1B). In contrast, Rab25 expression in HEY cells significantly reduced TRAIL-induced cell death (Figure 1C). Gene expression analysis (Supplementary Table 1) did not demonstrate detectable changes in the expression of death receptors (DR4 and DR5), decoy receptors (DcR1 and DcR2), and downstream signaling molecules proposed to mediate the action of TRAIL but not FasL in cells expressing Rab25. In agreement with the gene expression data, on western blotting DR4 and DR5 protein levels were not altered by Rab25 expression (Supplementary Figure 1).

OPG, a soluble decoy receptor for RANKL, also binds TRAIL as a decoy receptor and blocks its ability to activate death receptors [20]. Hence, we measured the effect of Rab25 on levels of OPG in culture media. Strikingly, secreted OPG levels were significantly higher in Rab25 expressing cells (Figure 1D), while down regulation of Rab25 expression by Rab25-specific RNAi decreased OPG levels, suggesting that Rab25 expression could either increase OPG production and/or release. Similar to the observations from HEY cells, expression of Rab25 in IOSE80ht and SKOV3 reduced cell death induced by TRAIL, but not by FasL (Supplementary Figure 2). Thus the ability of Rab25 to increase release of OPG could result in inactivation of TRAIL and decreased TRAIL-induced cell death in Rab25 expressing cells. Indeed, addition of 100 ng/ml of exogenous OPG to the culture media significantly reduced cell death induced by TRAIL in HEY cells transfected with empty vector, while only modest and non-significant effects were observed in Rab25 expressing HEY cells, which already produced high levels of endogenous OPG (Figure 1E). Consistent with the lack of effect of Rab25 on FasL-induced death, addition of exogenous OPG did not block FasL-induced cell death suggesting that OPG neutralizes TRAIL but not FasL action consistent with the ability of OPG to bind TRAIL and not FasL (Figure 1E).

### Rab25 regulates both OPG expression and secretion

The elevated OPG levels in culture media from Rab25 expressing cells could be due to increased transcription and translation, altered protein stability and/or increased secretion. We have recently reported the mRNA expression profile of A2780 cells expressing Rab25 (GSE28299) [24]. Based on this dataset, Rab25 induced a 1.7 fold increase in OPG mRNA expression. To further evaluate the relationship between Rab25 and OPG mRNA expression, we examined OPG mRNA levels in four addition cell lines stably expressed Rab25 [23]. Although all of the Rab25 expressing cell lines demonstrated increased OPG in culture supernatants (Figure 1D), mRNA levels as assessed by transcriptional profiling were only increased in HEY and MCF7 cells (Supplementary Table 2). Thus in a subset of cell lines that release OPG into the media, Rab25 is able to increase mRNA levels.

**Figure 2:**
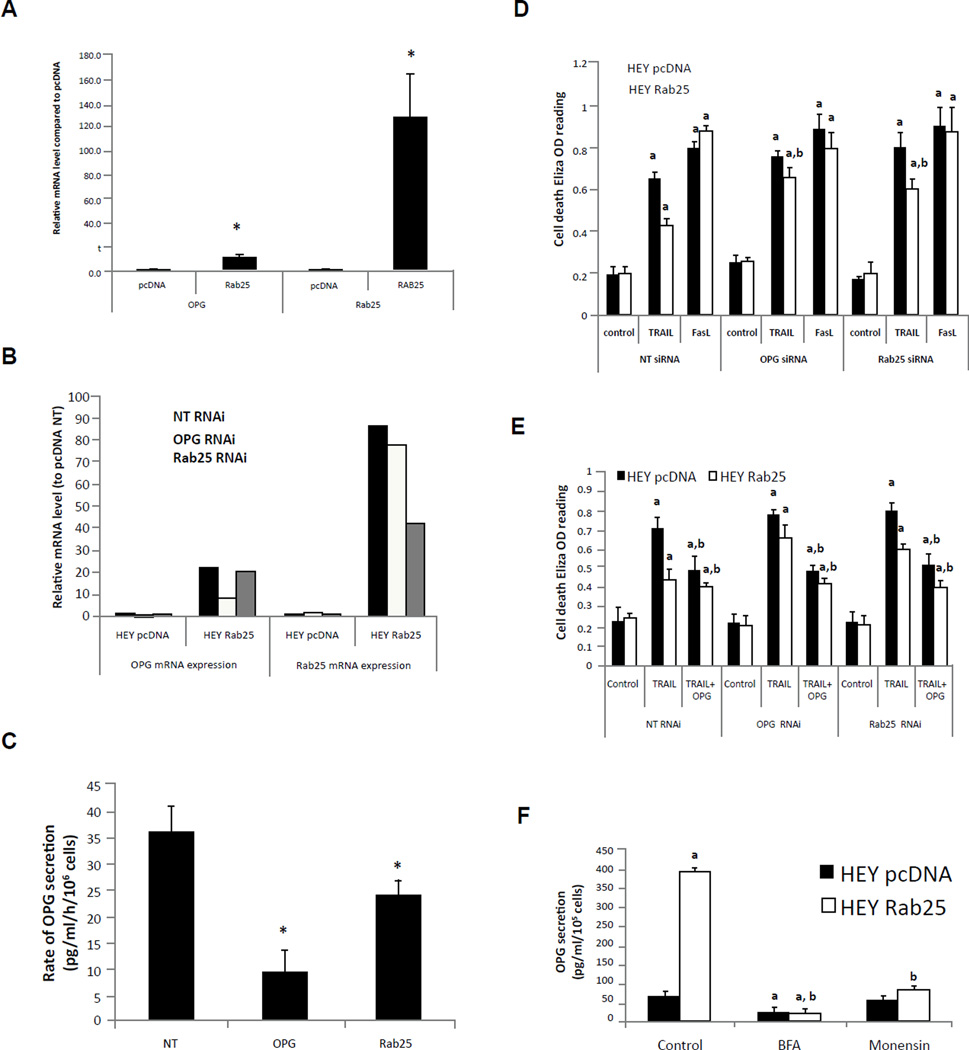
Rab25 regulates OPG expression. A) QPCR measurement of OPG and Rab25 mRNA level in HEY cells. *, p < 0.001 vs pcDNA control. (B) Effect of siRNA on OPG and Rab25 mRNA expression. Ovarian HEY cells were transfected with siRNA specific to OPG or Rab25, as well as non-target siRNA (NT) control. Samples were collected 24h post transfection and mRNA expression level was detected with QPCR (data are from one of three representative experiments). (C) Reduction of OPG production after siRNA knockdown expression of OPG and Rab25. OPG secretion in media was measured using Human Osteoprotegerin (OPG)/TNFRSF11B DuoSet purchased from R&D Systems. p < 0.05 vs NT siRNA control. (D) Knockdown expression of OPG and Rab25 by siRNA increase TRAIL sensitivity. Cells were transfected with siRNA specific to OPG, Rab25 or non-target siRNA (NT) for 24h, then media were changed in the present of 100ng/ml of TRAIL for 24h before assay. a, p < 0.01 vs no drug NT siRNA transfected control, b p < 0.05 vs NT siRNA transfected TRAIL treated samples. E) Addition of 100ng/ml of exogenous OPG blocks 100ng/ml TRAIL-induced cell death. a, p < 0.05 vs no drug treatment. b, p < 0.05 vs 100 ng/ml TRAIL treatment only. (F) Pretreatment cells with 10mM of Brefeldin A (BFA) or Monensin block OPG secretion. a, p < 0.01 vs HEY pc DNA control, b, p < 0.001 vs HEY Rab25 control.

As assessed by qPCR, Rab25 increased OPG mRNA levels in HEY cells (Figure 2A) consistent with the ability of Rab25 to increase OPG transcript levels (Supplementary Table 2). Although OPG and Rab25 mRNA levels are low in pcDNA expressing HEY cells (Figure 2A), they were effectively decreased by OPG RNAi and Rab25 RNAi, respectively (Supplementary Figure 3). However, as OPG levels in pcDNA expressing cells are below the detection limit of the OPG assay, these siRNAs did not detectably alter OPG production (data not presented). In contrast, siRNA knock down of OPG in Rab25 expressing HEY cells, effectively decreased both OPG mRNA levels (Figure 2B) and OPG release into culture media (Figure 2C). In Rab25 expressing HEY cells, Rab25 siRNA only modestly decreased Rab25 mRNA levels with Rab25 mRNA levels in siRNA treated cells still being much higher than Rab25 levels in parental HEY cells (Figure 2B). The degree of knockdown of Rab25 by Rab25 siRNA in Rab25 expressing HEY cells was not sufficient to decrease OPG mRNA levels (Figure 2B); however, it was sufficient to modestly decrease OPG protein levels in cell supernatants (Figure 2C). Thus Rab25 may independently regulate OPG mRNA levels and OPG secretion. The decrease of OPG in the culture media induced by OPG and Rab25 siRNA resulted in an increased sensitivity toward TRAIL-induced cell death, whereas there was no effect on FasL induced cell death (Figure 2D). Further, addition of exgenous OPG to the OPG and Rab25 RNAi transfected HEY cells reduced the degree of cell death induced by exogenously added TRAIL (Figure 2E), supporting OPG as a mediator of the ability of Rab25 to inhibit TRAIL induced death.

The majority of secretory proteins in eukaryotic cells share a common biosynthetic and secretion process with an origin in the rough endoplasmic reticulum (RER), followed by transport to the Golgi complex. In the trans-Golgi network (TGN), proteins destined for secretion are sorted and directed to the classical secretory pathway [26]. As seen in Figure 2F, OPG secretion was inhibited by monensin that disrupts the protein translocation within TGN or BFA that inhibits protein transport from the ER to the Golgi. As knock down of Rab25 expression decrease OPG secretion (Figure 2C) and monensin can trap OPG in the golgi apparatus (Figure 2F), Rab25 likely plays a role in trafficking OPG from the Golgi to cell membrane. Together, our data suggest that depending on the cell context, Rab25 can both increase transcription and faciltate release of OPG from the cell.

**Figure 3:**
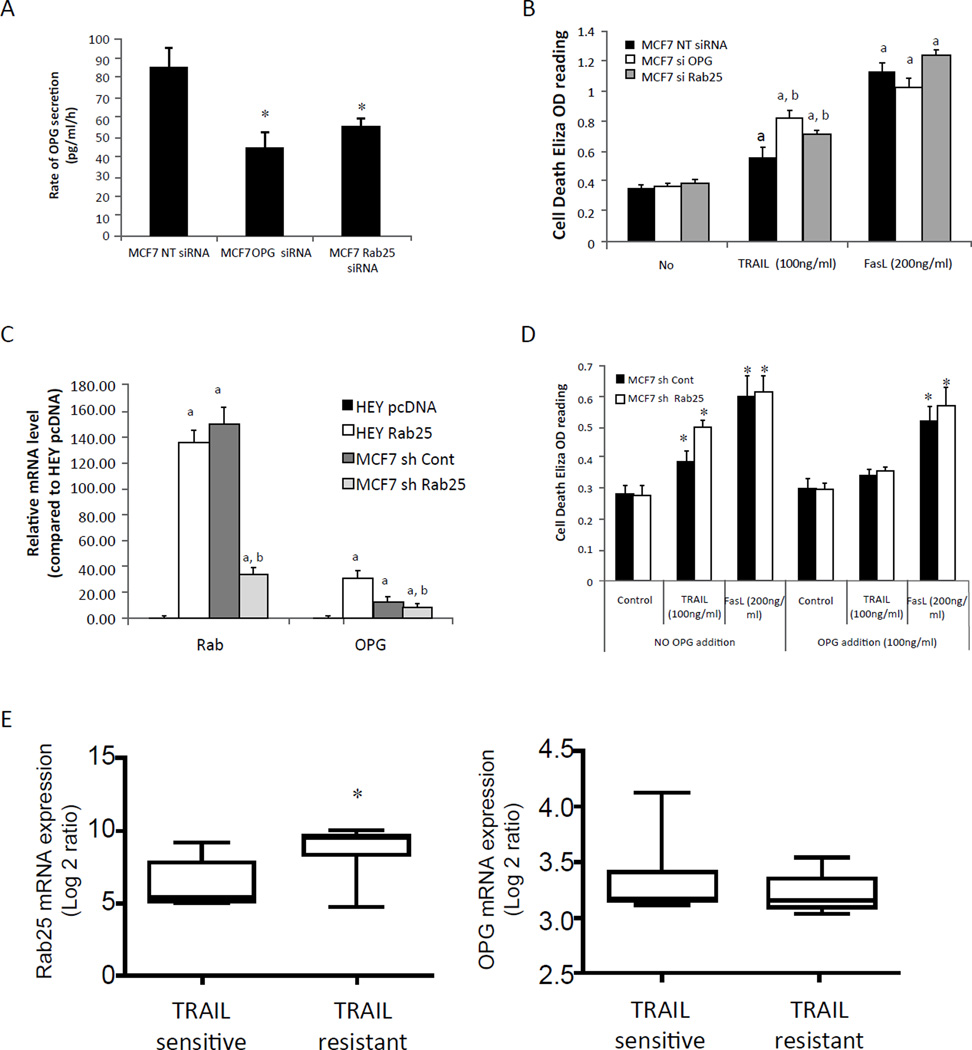
Effect of OPG in breast cancer MCF7 cells. (A) SiRNA knockdown OPG and Rab25 expression in MCF7 decrease OPG secretion. MCF7 cells were transfected with siRNA specific to OPG, Rab25 or non-target siRNA (NT) control. Flesh culture media was placed 24h post-transfection and OPG concentration was measured by Human Osteoprotegerin (OPG)/TNFRSF11B DuoSet purchased from R&D Systems*, p < 0.05 vs NT siRNA control. (B) TRAIL induced cells death was augmented by decreasing expression of OPG and Rab25 in MCF7 cells. a, p < 0.01 vs no drug NT siRNA transfected control, b p < 0.05 vs NT siRNA transfected TRAIL treated samples. (C) QPCR detection of OPG and Rab25 mRNA level in MCF7 Rab25 stably knockdown cells (MCF7 sh Rab25). The OPG and Rab25 expression level in HEY pcDNA was set to 1 for comparison. a, p < 0.01 vs HEY pc DNA control, b, p < 0.05 vs MCF7 shRNA control (MCF7 sh Cont). (D) Addition of exogenous OPG decreased MCF7 cells sensitivity to TRAIL-induced cell death. *, p < 0.05 vs no TRAIL control. (E) OPG and Rab25 mRNA expression in breast cancer cell line with respect to TRAIL sensitivity. *, p < 0.05 vs TRAIL sensitive cells.

To assess the generalizability of the role of Rab25 in regulating OPG and protecting cells from the effects of TRAIL, we utilized MCF7 breast cancer cells as a model, as they express both Rab25 and OPG (Figure 1D, Supplementary Table 1 and 2). Similar to the results obtained with HEY cells, transient transfection of MCF7 cells with Rab25 siRNA decreased Rab25 mRNA levels, but not OPG mRNA levels (data no shown). Again consistent with HEY cells, Rab25 knockdown in MCF7 cells decreased OPG levels in the media (Figure 3A), which was associated with an increase in sensitivity to TRAIL-induced cell death (Figure 3B). Importantly Rab25 knockdown did not alter the sensitivity of MCF7 cells to FasL-induced cell death. In support of the role of Rab25-mediated OPG expression in the protection of MCF7 cells from TRAIL-induced cell death, stable Rab25 shRNA knockdown (MCF7 shRab25) cells showed a significant decrease of Rab25 mRNA level as well as a slight reduction in total OPG mRNA levels (Figure 3C). As expected from the siRNA data (Figure 3A), stable knock down of Rab25 expression resulted in a reduction of OPG levels in culture media (Figure 1D). Importantly, Rab25 shRNA increased sensitivity to TRAIL- but not FasL-induced cell death (Figure 3D). Critically, addition of exogenous OPG (100ng/ml) to the culture media decreased TRAIL-induced cell death in the Rab25 shRNA expressing MCF7 cells. Thus in MCF7 and likely in HEY cells, Rab25 appears to primarily decrease OPG trafficking and release. Furthermore, the Rab25-mediated release of OPG protects cells from the effects of TRAIL but not FasL.

We analyzed the expression of OPG and Rab25 mRNA levels in 18 breast cell-lines with known sensitivity to TRAIL [27]. As seen in Figure 3E, TRAIL-resistant cells had higher Rab25 mRNA levels than TRAIL-sensitive cells consistent with a role for Rab25 in resistance to TRAIL. Interestingly, TRAIL-resistant cells also had modestly increased TRAIL mRNA levels (Supplementary Figure 4). However, OPG mRNA levels were modestly decreased in TRAIL-resistant cells (Figure 3E). Hence, resistance to TRAIL across breast cancer cell lines could potentially be due to Rab25-induced OPG secretion as seen in our HEY and MCF7 models.

### Signal transduction pathway regulates OPG expression

**Figure 4:**
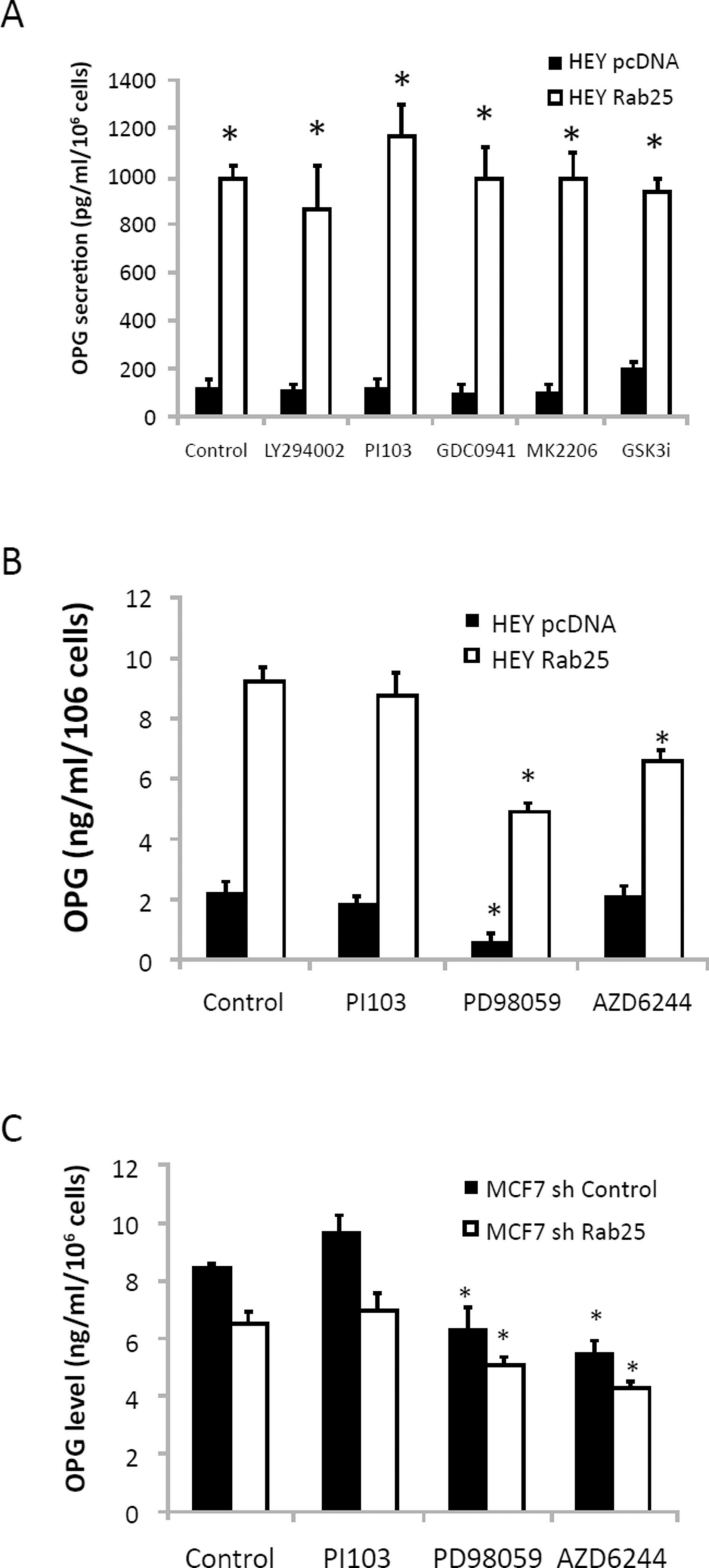
Signal transduction pathways regulation of OPG expression. (A) Effect of PI3K-AKT pathway inhibitor in ovarian HEY cells OPG secretion. Cells were treated with PI3K inhibitors (10mM of LY294002 or PI103), AKT inhibtor (10mM of GDC0941 or MK2206), or 10mM GSK3 inhibitor (GSKi) SB26763 for 24h before media collection; *, p < 0.05 vs pcDNA control). Effect of MAPK pathway inhibitor in OPG secretion in ovarian HEY (B) or MCF7 (C) cells. Cells were treated with MEK inhibitors (10mM of PD98059 or AZD6244). *, p < 0.05 vs no drug control.

AKT has been implicated in exocytosis/secretion and we have previously reported that Rab25 activates the AKT signal transduction pathway, at least in part, by directly binding AKT [23, 24]. To examine the role of the phosphatidylinositol 3 kinase (PI3K)/AKT pathway in Rab25-mediated OPG expression, we measured the effect of PI3K/AKT pathway inhibition on OPG secretion in HEY cells expressing Rab25 based on the high level of OPG production by these cells (Figure 1D). As seen in Figure 4A, PI3K-AKT inhibitors, including PI103, LY294002, GDC0941, MK2206, and SB216763, did not alter OPG secretion indicating that PI3K/AKT pathway activity is not required for the ability of Rab25 to increase OPG release. In contrast, OPG release was significantly reduced by MEK inhibitors including PD98059 and AZD6244, indicating that MAPK pathway activity is required for Rab25 to regulate OPG production (Figure 4B). In terms of generalizability the MEK/MAPK pathway inhibitors, but not the PI3K inhibitor, PI103, decreased OPG release from MCF7 cells (Figure 4C).

### EGF stimulates OPG expression

**Figure 5:**
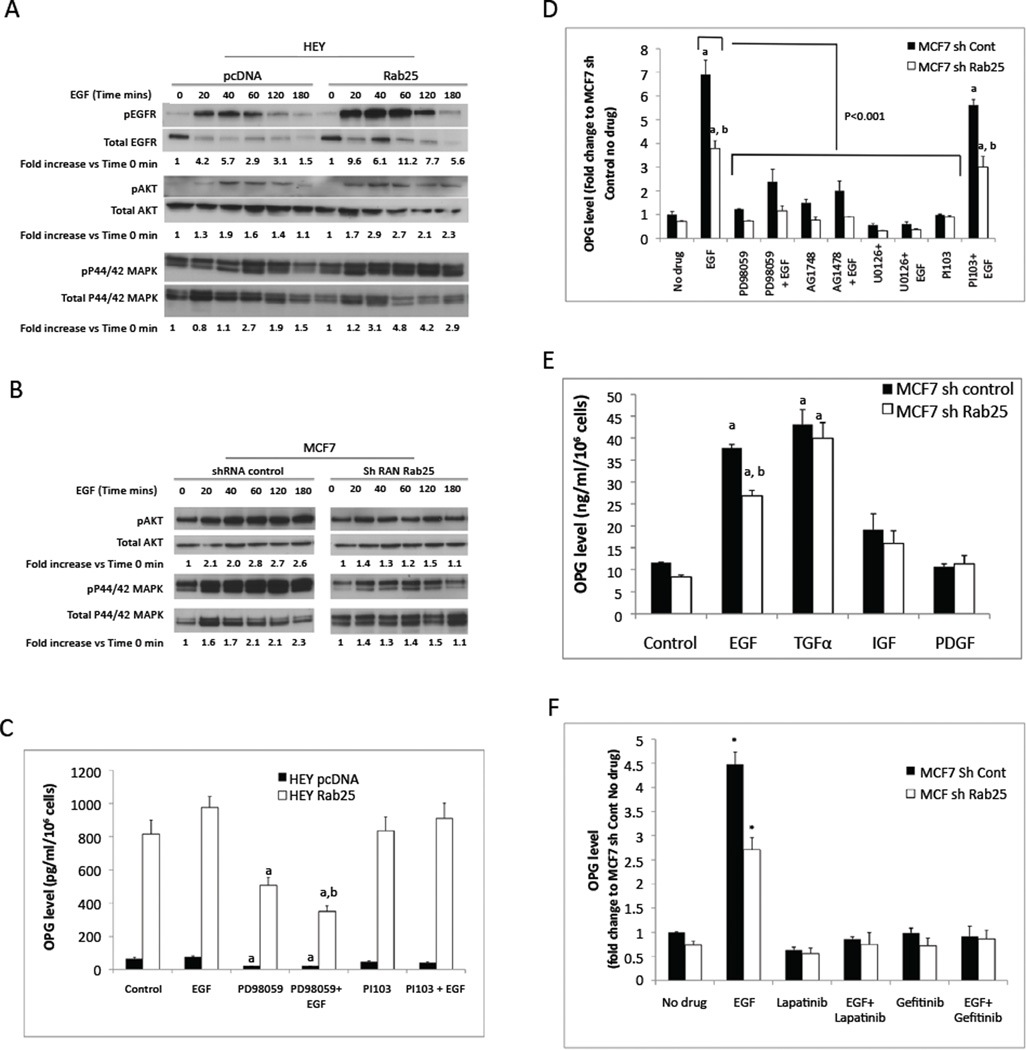
Rab25 expression enhances EGF activation of AKT and MAPK pathways. (A) Western blotting analysis of EGFR, AKT and MAPK activation after EGF stimulation. Ovarian HEY Cells stable Rab25 expression and its control cells (pcDNA) were treated with 100ng/ml EGF for indicated time before protein isolation. Fold increase in phosphorylated protein (compared to 0 min EGF stimulation) after normalization of total protein was shown below the figure. (B) Decrease activation of AKT and MAPK signal transduction in MCF7 cells stable knockdown Rab25. (C) MAPK inhibitor PD98059 (10mM) but not by PI3K inhibitor PI103 (10mM) blocked OPG expression. a, p < 0.05 vs no stimulation control; b, p < 0.05 vs PD98059 alone. (D) Inhibition of MAPK signal transduction abolished EGF stimulation of OPG secretion in MCF7 cells. MCF7 shRNA control or Rab25 stably knockdown cells (shRab25) were pretreated with 10mM of PD98059, 1mM of AG1478 or 1mM of U0126 for 60min before addition of 100 ng/ml of EGF. a, p < 0.01 vs no stimulation control; b, p < 0.01 vs MCF7 sh Control. (E) Stimulation of OPG secretion by 100ng/ml of EGFR ligands. a, p < 0.01 vs no stimulation control; b, p < 0.01 vs MCF7 sh Control. (F) Inhibition of EGF stimulated OPG secretion by EGFR inhibitors. MCF7 cells were pre-treated with either 1mM of Lapatinib or Gefitinib for 60min before addition of 100ng/ml EGF. Media concentration was measured 24h posted addition of EGF. *, p < 0.05 vs no drug control.

EGF, which activates both AKT and MAPK pathways, plays an important role in both ovarian and breast cancer pathophysiology [28]. Indeed, EGF increased both AKT and MAPK phosphorylation in HEY cells Rab25 expression increased both the duration and magnitude of EGFR signaling (Figure 5A). In agreement with the prolonged activation of EGFR in Rab25 expressing HEY cells, down-regulation of Rab25 expression in MCF7 cells reduced both the magnitude and duration of EGF-induced AKT and MAPK phosphorylation (Figure 5B). EGF modestly if at all increased secretion of OPG by HEY cells with or without Rab25 expression (Figure 5C). However, in both the presence and absence of EGF, PD98059, but not PI103, decreased OPG release (Figure 5C). In contrast to HEY cells, EGF markedly enhanced OPG secretion by MCF7 cells (Figure 5D). Knock down of Rab25 diminished effect of EGF on OPG accumulation in cell media (Figure 5D). Again, the effect of EGF on OPG secretion was mediated through the MAPK pathway as addition of MEK inhibitors, including PD98059, AG1478 and U0126, essentially reversed the effect of EGF on OPG secretion (Figure 5D). In contrast, blocking the PI3K pathway did not alter OPG production induced by EGF. Interestingly, TGFα, gand, but not IGF1 or PDGF, increased OPG release by MCF7 cells independent of Rab25 levels (Figure 5E). As expected, EGF-induced OPG release is dependent on EGFR activation as both Lapatinib and Gefitinib, abolished the effect of EGF on OPG release (Figure 5F).

### Inhibition of the EGFR enhances TRAIL sensitivity

**Figure 6:**
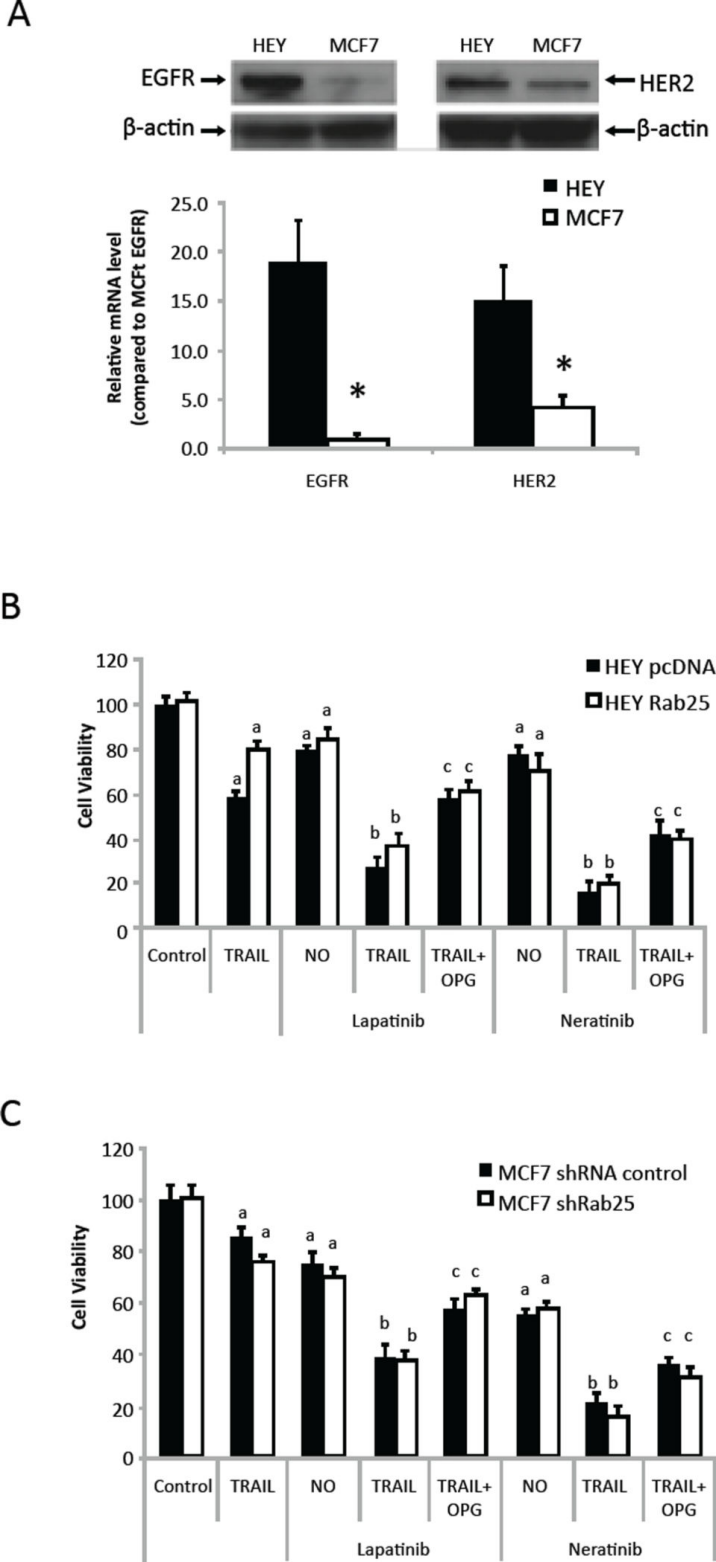
EGFR inhibitor promotes cell death induction by TRAIL. (A) Expression of EGFR and ERBB2 in HEY and MCF7 cells was detected by qPCR and western blotting analysis. EGFR level in MCF7 cells was set to 1 for comparison; *, p < 0.05 HEY vs MCF7 cells. EGFR inhibitors increased cell sensitivity to TRAIL-induced cell death in ovarian HEY (B) and breast MCF7 (C) cells. Cells were pre-treated with either 1mM of Lapatinib or Neratinib for 8h before addition of TRAIL in the present or absent of OPG (100ng/ml). a, p < 0.05 vs no drug control; b, p < 0.05 vs TRAIL only without EGFR inhibitor; c, p < 0.05 vs TRAIL alone in the present of Lapatinib or Neratinib.

Since inhibition of EGFR signaling decreased OPG production (Figure 5), we tested whether pan-EGFR family inhibitors, Lapatinib and Neratinib, could alter effects of TRAIL in HEY and MCF7 cells that express both EGFR and HER2, as detected by qPCR and western blotting analysis (Figure 6A). As shown in Figures 6B and 6C, pre-treating HEY and MCF7 cells with either Lapatinib or Neratinib enhanced cell death induced by TRAIL independent of the presence or absence of Rab25. Again, in agreement with the role of OPG as an inhibitor of TRAIL-induced cell death, addition of exogenous OPG (100ng/ml) reduced the sensitivity of HEY and MCF7 cells to TRAIL.

## Discussion

Rab small G proteins represent the largest family of the Ras superfamily of monomeric G proteins, with over 60 mammalian gene products. Rabs play essential roles in all stages of vesicle trafficking including internalization, targeting and cargo selection [29]. The Rab11 subfamily, consists of Rab11a, Rab11b and Rab25 (aka Rab11c/CATX), are involved in regulating recycling of internalized membrane proteins and movement of membrane proteins between polarized surfaces of epithelial cells [30]. To date several Rab GTPase family members have been shown to participate in regulating cellular secretion including members of the Rab11 subfamily [31]. Rab11a has recently been identified to play a role in cytokine release [32], in addition to its canonical role in regulating apical vesicle recycling [33]. Similarly, Rab11b regulates insulin secretion [34]. Unlike Rab11a and 11b, Rab25 is less well characterized and the endogenous cargoes for Rab25-dependent trafficking remain generally unclear. In the present study, we demonstrated that Rab25 plays a role in regulating OPG release providing a survival advantage for cancer cells in the presence of TRAIL.

OPG binds TRAIL produced by tumor infiltrating monocytes, with high affinity [20]. TRAIL binds and induces apoptosis of tumors cells while having modest effects on normal cells [35] making it a potential therapeutic option. Preclinical studies in mice demonstrated that recombinant TRAIL suppresses the growth of multiple human tumor xenografts with no apparent systemic toxicity [10]. Recently, recombinant TRAIL has entered clinical trials for the treatment of cancer [11, 36]. Although phase 1 and 2 studies have indicated tolerable levels of toxicity, therapeutic efficiency was unfortunately disappointing [37]. It is now known that not all tumor cells are sensitive to TRAIL despite the expression of the death receptors on the surface [38]. Resistance to TRAIL has been proposed to occur through multiple mechanisms including: low expression or loss of function of TRAIL-R1 and -R2, increased levels of DcR1 or DcR2, elevated levels of negative regulators of apoptosis such as cFLIP, upregulation of cell survival and proliferation pathways, through mitogen-activated protein kinases (MAPK) and nuclear factor-κB (NF-κB) activation, and production of OPG [13–16, 39]. Our data demonstrated that Rab25 expression to levels found in human tumors with *RAB25* amplification, which is associated with aggressiveness of breast and ovarian cancer [23], increases OPG levels in the supernatant of cancer cells. The release of OPG is sufficient to inhibit TRAIL-induced apoptosis. In addition to its function in antagonizing TRAIL-mediated apoptosis, recent studies have demonstrated that OPG can promote cancer and endothelial cell survival independent of anti-TRAIL effect and also induce angiogenesis [18]. Further OPG synthesized and released from breast cancer cells exhibits pro-metastatic activity and promotes bone specific colonization potential, which is independent of its anti-TRAIL and RANKL activity [18]. Hence, the ability of Rab25 to increase OPG expression in a subset of cancer cell lines and to increase OPG release by the majority of cancer cell lines assessed, suggests that amplification of Rab25 may provide survival advantages for cancer cell independent of TRAIL and RANKL. Indeed, OPG serum levels have been reported to be significantly higher in patients with advanced cancer and those with cancer metastatic to bone [18]. In addition, recent studies have detected the expression of OPG in ovarian cancer patient ascites [40], which protected the ovarian cancer cells from TRAIL-induced cell death [41]. Whether elevated Rab25 levels due to chromosome 1q amplification contributes to the elevated OPG levels in ascites of ovarian cancer patients remains to be determined.

EGF has been reported to block TRAIL-induced apoptosis through activation of AKT and subsequent inhibition of cytochrome c release, downstream caspase 8 activation and cleavage of BID [42]. However, in the cells studied, herein, the major pathway involved in OPG release appears to be due activation of the MEK/MAPK pathway. Both positive and negative effects of EGF mediated EGFR activation on OPG expression have been reported with, EGF stimulating OPG expression in prostate LNCaP cells [43], but inhibiting OPG expression in oesteoblastic cells [44]. We demonstrated that EGF increased OPG production particularly in cells with high levels of Rab25. The prolonged activation of the AKT and MAPK pathways induced by EGF when Rab25 levels are elevated may be due to increased Rab25 mediated EGFR recycling to the membrane [29]. However, this also renders Rab25-expressing cells more susceptible to pan-EGFR family inhibitors as higher cell death was induced by TRAIL in the presence of Lapatinib or Neratinib [45]. EGFR inhibitors also increased TRAIL-induced apoptosis in lung [28] and bladder [46] cancer cells suggesting that this may be a generalizable process. Chemotherapeutic agents and/or radiotherapy have been found to restore or enhance TRAIL sensitivity in a range of tumors including breast [47], prostate [48] and lung cancers [49], and in a number of cases a synergistic effect could be achieved.

In summary, increased OPG release in the presence of high endogenous Rab25 levels may provide a survival advantage for cancer cells and contribute to selection of tumors with elevated Rab25 levels [23]. Rab25 may increase OPG production, at least in part, through increasing responsiveness to EGFR ligands. Whether a combination of TRAIL with EGFR inhibitors will be effective in patients with tumors expressing high levels of Rab25 warrants further investigation.

## Supplementary Material

Supplementary File
