# Supplementary material for: Rab25 Small GTPase Mediates Secretion of Tumor Necrosis Factor Receptor Superfamily Member 11b (osteoprotegerin) Protecting Cancer Cells from Effects of TRAIL
Source: J Genet Syndr Gene Ther. Author manuscript; Available in PMC 2014 Dec 15. (PMC4266180; doi:10.4172/2157-7412.1000153)
Supplement: Supplementary File [file NIHMS606685-supplement-Supplementary_File.docx]

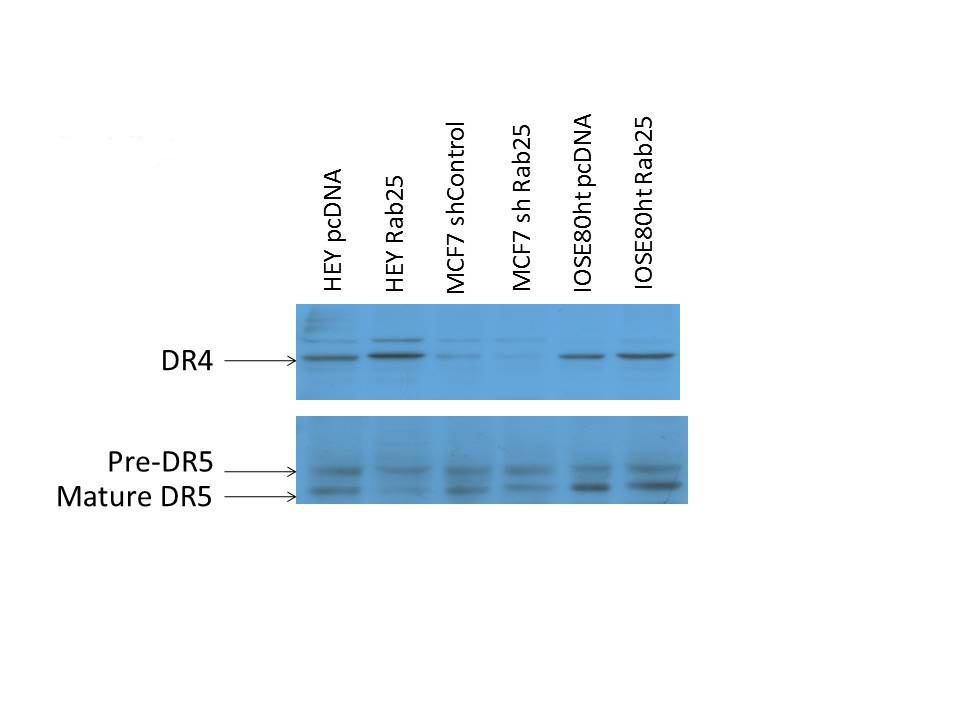


**Supplementary Figure 1**: Western blotting analysis of TRAIL receptor , DR4 and DR5, expression


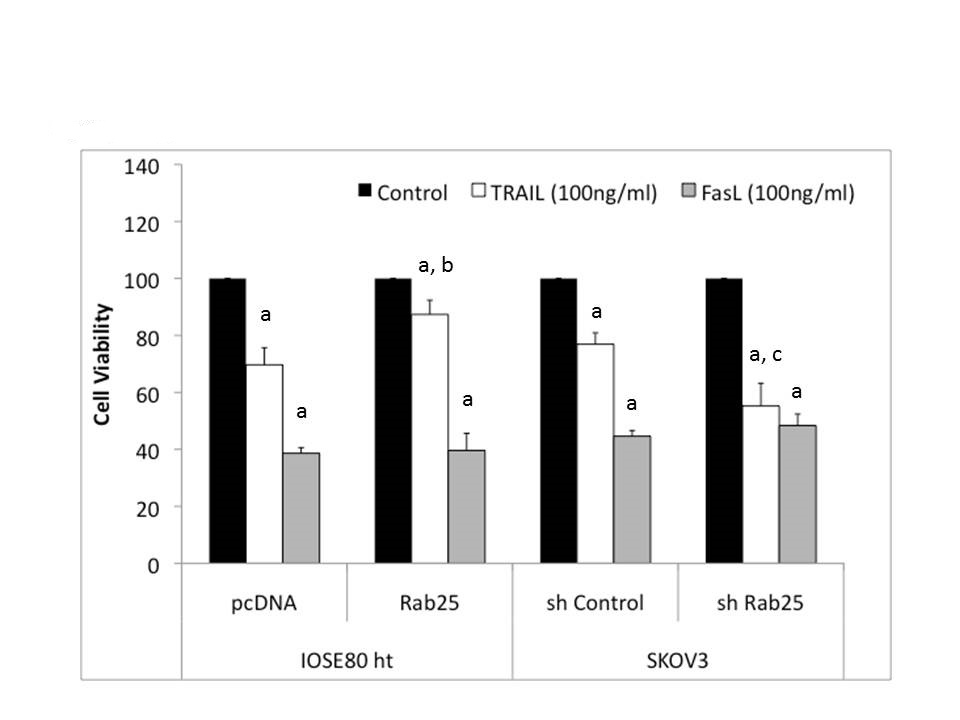


**Supplementary Figure 2**: Rab25 expression alters cell sensitivity to TRAIL induced cell death. Cells were treated with 100 ng/ml of FasL or TRAIL for 24h before detection of cell viability using Cell-Titer Blue assay. a, p < 0.05 vs no drug control; b, p < 0.05 Rab25 vs pcDNA in TRAIL treated IOSE80ht cells; c, p < 0.05 shRab vs sh Control in TRAIL treated SKOV3 cells.


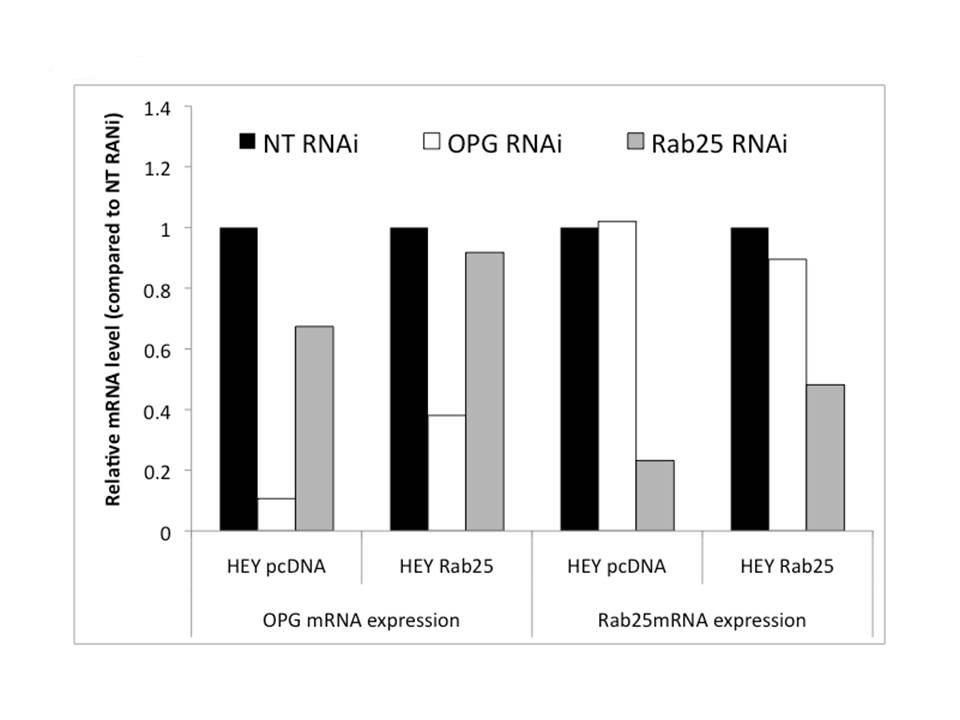


**Supplementary Figure 3:**Effect of siRNA on OPG and Rab25 mRNA expression. Ovarian HEY pcDNAcells were transfected with siRNAspecific to OPG or Rab25, as well as non-target siRNA (NT) control. Samples were collected 24h post transfection and mRNA expression level was detected with QPCR. The expression level in NT RNAi cells was set to 1 for comparison (data are from one of three representative experiments).

**
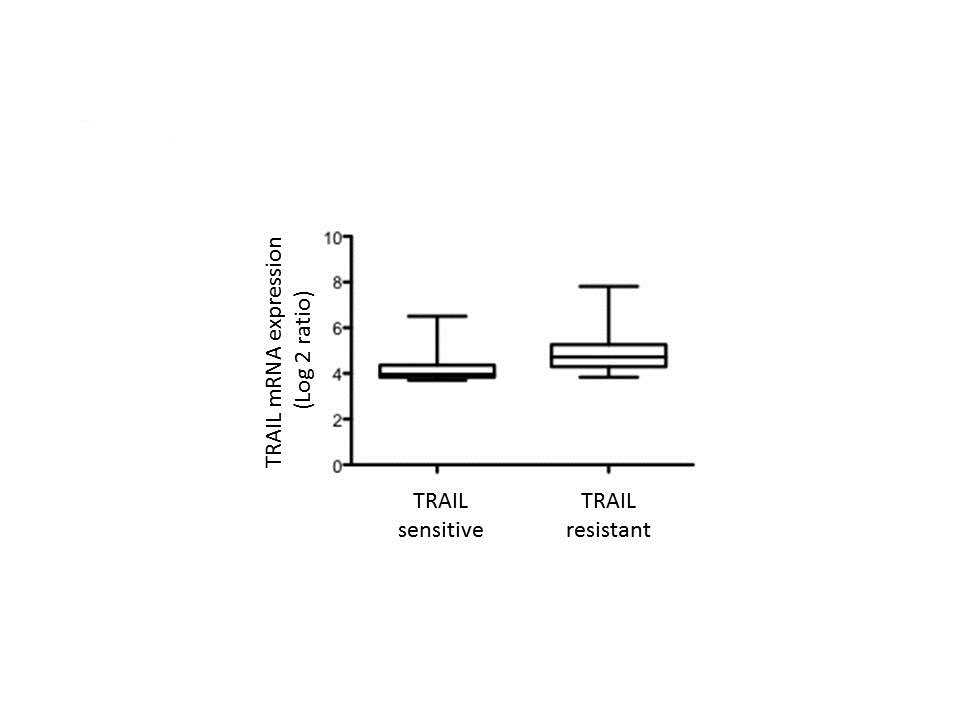
**

**Supplementary Figure 4**: TRAIL mRNA expression in breast cancer cell line with respect to TRAIL sensitivity. *, p < 0.05 vs TRAIL sensitive cells.
